# On the Value of Considering Specific Facets of Interactional Justice Perceptions

**DOI:** 10.3389/fpsyg.2020.00812

**Published:** 2020-05-15

**Authors:** Evelyne Fouquereau, Alexandre J. S. Morin, Tiphaine Huyghebaert, Séverine Chevalier, Hélène Coillot, Nicolas Gillet

**Affiliations:** ^1^EE 1901 QualiPsy, Département de Psychologie, Université de Tours, Tours, France; ^2^Substantive Methodological Synergy Research Laboratory, Department of Psychology, Concordia University, Montreal, QC, Canada; ^3^Laboratoire C2S, Département de Psychologie, Université de Reims Champagne-Ardenne, Reims, France

**Keywords:** organizational justice, latent profiles, bifactor, confirmatory factor analyses, transformational leadership, well-being, burnout

## Abstract

This research seeks to verify the value of considering specific perceptions of informational and interpersonal justice over and above employees’ global perceptions of interactional justice. In Study 1 (Sample 1: *n* = 592; Sample 2: *n* = 384), we examined the underlying structure of workers’ perceptions of interactional justice by contrasting first-order and bifactor representations of their ratings. To investigate the true added value of specific informational and interpersonal justice perceptions once global interactional justice perceptions are taken into account, we also considered the relations between these global and specific perceptions and various outcomes. Our findings revealed that workers’ perceptions of interactional justice simultaneously reflected a global interactional justice factor and two specific facets (interpersonal and informational justice). In Study 2, we identified employees’ latent justice profiles based on their global (interactional justice) and specific (interpersonal and informational justice) levels of interactional justice. Five different interactional justice profiles were identified: *low interpersonal*, *high interpersonal/average informational*, *high informational*, *normative*, and *high interpersonal/low informational*. Employees’ perceptions of transformational leadership are a significant predictor of profile membership. Finally, the five profiles were significantly associated with anxiety and emotional exhaustion.

## Introduction

Numerous studies in the organizational and managerial literature (Colquitt et al., 2001) have been conducted to examine workers’ perceptions of organizational justice (i.e., the extent to which they are fairly treated by organizational authorities; Colquitt, 2001). Unfortunately, the distributive (i.e., the fairness linked to the distribution of resources and rewards) and procedural (i.e., the fairness linked to organizational procedures and processes) facets of organizational justice (Colquitt, 2001) are not easy to directly influence by managers, and their optimization often requires organization-wide interventions (Rineer et al., 2017). However, this is not the case for the interactional facet of organizational justice (i.e., the extent to which workers perceive that their managers treat them with truthfulness, respect, and dignity; Scott et al., 2009). Interactional justice is characterized by the two subcomponents of informational (i.e., providing adequate explanations for decisions and procedures and discussing them with honesty) and interpersonal (i.e., treating employees with respect while refraining from prejudicial or improper statements when interacting with them) justice (Colquitt, 2001). Nevertheless, interactional justice is also represented by a single overarching dimension (Ambrose et al., 2013; Buengeler and Den Hartog, 2015). The identification of high correlations between informational and interpersonal justice (Kass, 2008) and the fact that a second-order factor of interactional justice is more strongly to predictors and consequences than its first-order components (e.g., Rego and Pina e Cunha, 2010) both suggest that a global approach may be appropriate. Yet, research also shows differentiated patterns of associations between these two components with a variety of external criteria (Au and Leung, 2016).

These observations raise an important question related to whether interpersonal and informational justice really retains sufficient specificity once global interactional justice is considered (Morin et al., 2017). The current research specifically addresses this question by relying on a combination of variable- and person-centered approaches specifically designed to investigate the dimensionality of psychological constructs (Morin et al., 2017). More precisely, we first rely on variable-centered bifactor measurement models to directly analyze the multidimensionality of the interactional justice construct and whether this measurement structure will generalize to a new independent sample of participants (Study 1). In addition, these two studies were also designed to better document the relative role of the global (interactional) and specific (interpersonal and informational) components in the prediction of outcomes related to employee-relevant (Sample 1: positive and negative affect, life satisfaction, and burnout components) and organization-relevant (Sample 2: commitment and organizational citizenship behaviors) outcomes. In a second study, we complement these variable-centered results by relying on person-centered analyses to identify employees’ interactional justice perception profiles, while considering one predictor (transformational leadership) and two outcomes (anxiety and emotional exhaustion) of these profiles (Study 2).

### Coexisting Global and Specific Interactional Justice Components

Generally, researchers (e.g., Colquitt and Shaw, 2005) rely on second-order factor models (e.g., Rindskopf and Rose, 1988) in which each item is used to define first-order factors (interpersonal and informational justice), which themselves define a second-order factor (interactional justice). In contrast, bifactor models are characterized by one global (G) factor (interactional justice) underlying the answers to all items coupled with a series of orthogonal specific (S) factors (interpersonal and informational justice) with sufficient specificities (S factors) not explained by the G factor. The present research relies on a bifactor model in order to more precisely examine the dimensionality of employees’ interactional justice perceptions.

### The Joint Effects of Informational and Interpersonal Justice

Both informational justice and interpersonal justice are seen as essential for the well-being of employees (e.g., positive affect, life satisfaction) and for the emergence of desirable work outcomes (e.g., commitment to the organization) (Kass, 2008). In contrast, low interactional justice perceptions are associated with negative outcomes (e.g., negative affect, anxiety, burnout; Gillet et al., 2015). According to social exchange theory, perceptions of being treated fairly should encourage positive exchange relationships between employees and their managers, in turn leading to an increase in well-being and positive work behaviors among employees (Colquitt et al., 2001).

However, a limited number of studies have examined the combined effects of various justice components. Among the few studies on this topic, Loi et al. (2009) examined the effects of organizational justice on job satisfaction. The results showed that intraindividual fluctuations in daily perceptions of informational and interpersonal justice were positively related to daily variations in job satisfaction levels. In addition, interindividual differences in distributive justice perceptions moderated the within-person effects of interpersonal justice on job satisfaction. More specifically, this relation was stronger when distributive justice levels were low. Similarly, interindividual differences in procedural justice perceptions moderated the effects of informational justice on job satisfaction. More specifically, this relation was stronger when procedural justice levels were low.

Choi (2008) similarly showed that employees’ organizational justice perceptions moderated the positive effects of justice perceptions associated with specific events on organizational commitment and citizenship behaviors, such that these relations were stronger when levels of justice ascribed to the organization were low. Likewise, employees’ justice perceptions related to their supervisor moderated the positive relations between their justice perceptions associated with specific events and employees’ trust in, and citizenship behaviors directed at, their supervisor, such that the links were stronger when levels of justice ascribed to the supervisor were low. Similar results were reported by Brockner and Wiesenfeld (1996), who showed that distributive justice perceptions were less strongly related to employee outcomes when procedural justice perceptions were high, and vice versa. Finally, Skarlicki and Folger (1997) showed that interactional, procedural, and distributive justice interacted to predict organizational retaliation behaviors. More precisely, benefits were associated with distributive justice perceptions when both procedural and interactional justice perceptions were low. Similar compensatory mechanisms involving distributive and procedural justice perceptions were only observed when interactional justice perceptions were low, whereas those between interactional and distributive justice perceptions were only observed when procedural justice perceptions were low. Globally, these results suggest that different facets of organizational justice may have beneficial compensatory effects for employees exposed to low levels on other justice perceptions. Unfortunately, these studies did not consider the combined effects of informational and interpersonal justice perceptions or the possibly differentiated relations involving global interactional justice perceptions from those involving specific interpersonal and informational justice perceptions.

### A Person-Centered Perspective

Person-centered analyses are useful for examining the interactive and complementary effects of employees’ global perceptions of interactional justice with their specific levels of interpersonal and informational justice involving person-centered analyses. Researchers relying on person-centered analyses can identify distinct subpopulations (or profiles) of employees presenting different levels on these interactional justice components. Contrasting with variable-centered analyses that assume that all employees come from the same population, person-centered analyses [e.g., latent profile analyses (LPA)], are specifically designed to identify qualitatively distinct profiles of workers characterized by distinct configurations of interactional justice components (Morin, 2016). Latent profile analyses thus provide a complementary way to examine the combination of the facets of interactional justice for different profiles of workers. Unfortunately, no person-centered research has yet investigated interactional justice perceptions.

When global constructs (e.g., interactional justice) coexist with specific dimensions (i.e., informational and interpersonal justice), failure to consider the coexistence of these global and specific components in LPA may lead authors to identify similarly shaped profiles with differences on global interactional justice (i.e., referred to as level-differentiated profiles relative to shape-differentiated profiles). Therefore, researchers should test preliminary measurement models, extract factor scores, and estimate LPA based on these factor scores (Morin et al., 2017). In Study 2, we adopt the approach advocated by these authors.

## Study 1

In Study 1, we examined the structure of a measure of employees’ interactional justice perceptions and the replicability of those results across two independent samples of participants. Second, to document the value of considering employees’ specific interpersonal and informational justice perceptions over and above their global perceptions of interactional justice, we consider relations between these global and specific perceptions and employees’ (a) well-being (life satisfaction and positive affect) and ill-being (emotional exhaustion, physical fatigue, and cognitive weariness) at work in Sample 1; and (b) work attitudes (affective, normative, and continuance commitment to their organization) and behaviors (altruism and civic virtue) in Sample 2. We focus on these outcomes given evidence supporting their importance (Huang and Simha, 2018). Indeed, research has shown that well-being and ill-being are important predictors of work performance (Rofcanin et al., 2017) and turnover intentions (Caesens et al., 2016). Similarly, organizational commitment is strongly related to persistence, engagement, and work performance (von Bonsdorff et al., 2015). Finally, organizational citizenship behaviors are significantly related to numerous individual and organizational outcomes (Yang et al., 2016). Research generally shows that interactional justice tends to produce positive work-related outcomes and low levels of ill-being (e.g., Gillet et al., 2015). We thus expected global levels of interactional justice and the remaining specific interpersonal and informational justice S factors to be associated with lower levels of ill-being but higher levels of commitment, desirable work behaviors, and well-being.

### Methods

#### Participants and Procedures

Research assistants distributed a paper-based questionnaire to two distinct convenience samples of employees working in various French organizations (e.g., industries, services, sales, public hospitals). All participants were guaranteed that only aggregate data would be reported and provided informed consent.

Sample 1 included 592 workers (222 men, 370 women). Ages ranged from 19 to 62 years with a mean of 36.63 (SD, 11.99) years. A total of 478 participants were full-time workers (80.7%), whereas 500 participants were permanent workers (84.5%), and 92 were temporary workers (15.5%). Seventeen participants (2.9%) had no diploma, 138 had a vocational training certificate (23.3%), 130 had a high school diploma (22.0%), and 307 had a university diploma (51.9%).

Sample 2 included 384 workers (156 men, 228 women) aged between 18 and 62 years [mean, 38.53 (SD, 13.02) years], with an average organizational tenure of 11.19 (SD, 11.02) years and an average tenure in the current position of 6.85 (SD, 7.39) years. A large majority of participants were full-time workers (78.9%), whereas 318 participants were permanent workers (82.8%), and 66 were temporary workers (17.2%). This sample included 92 participants employed in the public sector (24.0%) and 292 employed in the private sector (76.0%). Twelve participants (3.1%) had no diploma, 92 had a vocational training certificate (24.0%), 126 had a high school diploma (32.8%), and 154 had a university diploma (40.1%).

#### Measures

##### Interactional justice (Samples 1 and 2)

Interpersonal justice (four items, α = 0.92 in Sample 1; α = 0.87 in Sample 2; e.g., “Your supervisor treats you in a polite manner”) and informational justice (five items, α = 0.91 in Sample 1; α = 0.88 in Sample 2; e.g., “Your supervisor explains the procedures thoroughly”) were assessed with the justice items proposed by Colquitt (2001). Items were rated on a seven-point Likert scale (1 = strongly disagree to 7 = strongly agree).

##### Positive and negative affect (Sample 1)

Ten items from the Positive and Negative Affect Schedule (Watson et al., 1988) were used to assess positive (five items; α = 0.60; e.g., “active”) and negative (five items, α = 0.69; e.g., “afraid”) affect. Items were rated on a five-point frequency response scale (1 = never to 5 = always).

##### Life satisfaction (Sample 1)

The Satisfaction With Life Scale (five items; α = 0.88; e.g., “The conditions of my life are excellent”; Diener et al., 1985) was used to assess life satisfaction. Items were rated on a seven-point Likert scale (1 = strongly disagree to 7 = strongly agree).

##### Burnout (Sample 1)

The Shirom–Melamed Burnout Measure (Shirom and Melamed, 2006) was used to assess emotional exhaustion (three items, α = 0.87; e.g., “I feel I am unable of being sympathetic to coworkers”), cognitive weariness (five items, α = 0.94; e.g., “I feel I am not thinking clearly”), and physical fatigue (six items, α = 0.92; e.g., “I feel physically drained”). Items were rated on a seven-point frequency scale (1 = never to 7 = always).

##### Commitment to the organization (Sample 2)

We used Bentein et al’s (2005) scales to assess affective (six items, α = 0.86; e.g., “I feel emotionally attached to this organization”), normative (six items, α = 0.89; e.g., “I think I would be guilty if I left my current organization now”), continuance: high sacrifice (three items, α = 0.77; e.g., “I would not leave this organization because of what I would stand to lose”), and continuance: low alternatives (three items, α = 0.69; e.g., “I feel that I have too few options to consider leaving this organization”) commitment to the organization. Items were rated on a five-point Likert scale (1 = strongly disagree to 5 = strongly agree).

##### Organizational citizenship behaviors (Sample 2)

Two subscales from Podsakoff and MacKenzie (1994) were used to assess altruism (two items, α = 0.81; “I willingly give of my time to help other agents who have work-related problems”) and civic virtue (three items, α = 0.84; “I attend and actively participate in company meetings”). Items were rated on a five-point Likert scale (1 = strongly disagree to 5 = strongly agree).

### Analyses

All models were estimated using robust maximum likelihood (MLR) estimator in Mplus 8 (Muthén and Muthén, 2017) and with full information maximum likelihood (FIML; Enders, 2010) to handle the missing responses at the item level (Sample 1: 0.00%–0.51%; Sample 2: 0.00%–1.30%). Confirmatory factor analyses (CFAs) and bifactor CFA representations of participants’ ratings of interactional justice were estimated and compared following Morin et al’. (2016, 2017) recommendations. As a baseline comparison model, we first estimated a simple one-factor CFA model (interactional justice) and a two-factor CFA model (interpersonal and informational justice). In bifactor CFA, all items were allowed to simultaneously load on one G factor reflecting global levels of interactional justice and on two S factors corresponding to specific levels of interpersonal and informational justice. Correlated CFA factors representing the outcomes were integrated to these models and specified as regressed on the justice factors.

We assessed the fit of the alternative models with the comparative fit index (CFI), the Tucker-Lewis index (TLI), and the root mean square error of approximation (RMSEA). Values greater than 0.90 and 0.95 for the CFI and TLI, respectively, are considered to be indicative of adequate and excellent fit to the data, whereas values smaller than 0.08 or 0.06 for the RMSEA, respectively, support acceptable and excellent model fit (e.g., Marsh et al., 2005). In the comparison of nested models, typical interpretation guidelines suggest that models differing by less than 0.01 on the CFI and TLI, or 0.015 on the RMSEA, can be considered to provide an equivalent level of fit to the data (Cheung and Rensvold, 2002). For complementary purposes, we also report the Bayesian information criteria (BIC), the sample-size adjusted BIC (ABIC), the Akaike information criteria (AIC), and the consistent AIC (CAIC). Although these information criteria do not provide information about the absolute fit of a model, lower values in model comparisons suggest a better-fitting model. For all models, we report standardized parameter estimates and composite reliability coefficients associated with the *a priori* factors, which are calculated from the model standardized parameters using McDonald (1970) omega: ω = (∑|λ_*i*_|)^2^/[(∑|λ_*i*_|)^2^ + ∑δ_*i*_], where |λ_*i*_| and δ_*i*_ are standardized factor loadings and item uniquenesses.

### Results

The goodness-of-fit indices of the measurement models are presented in Table 1 (Sample 1: top section; Sample 2: middle section). These results showed that, in both samples, an acceptable level of model fit was achieved both for the *a priori* two-factor CFA and bifactor CFA models, whereas the one-factor model failed to provide an even minimally acceptable level of model fit. In addition, these findings supported the superiority of the bifactor CFA model relative to that of the two-factor CFA model (Sample 1: ΔCFI = + 0.015; ΔTLI = + 0.013; ΔRMSEA = -0.013; Sample 2: ΔCFI = + 0.024; ΔTLI = + 0.023; ΔRMSEA = -0.019), a conclusion that was supported by the observation of lower values on all information criteria for the bifactor CFA solution.

**TABLE 1:** Goodness-of-fit statistics for the estimated measurement and predictive models.

Description	χ^2^ (df)	CFI	TLI	RMSEA	90% CI	AIC	CAIC	BIC	ABIC
**Study 1: Sample 1**									
M0. One-factor model	627.445 (27)*	0.774	0.698	0.194	[0.181; 0.207]	16,629	16,775	16,748	16,662
M1. Two-factor CFA	102.625 (26)*	0.971	0.960	0.071	[0.057; 0.085]	15,867	16,018	15,990	15,901
M2. Bifactor CFA	54.180 (18)*	0.986	0.973	0.058	[0.041; 0.076]	15,807	16,001	15,965	15,851
M3. M1 with outcomes	1,493.153 (637)*	0.929	0.921	0.048	[0.045; 0.051]	61,311	62,075	61,933	61,482
M4. M2 with outcomes	1,456.746 (623)*	0.930	0.921	0.048	[0.044; 0.051]	61,267	62,107	61,951	61,456
**Study 1: Sample 2**									
M0. One-factor model	329.230 (27)*	0.798	0.730	0.171	[0.154; 0.187]	9,288	9,422	9,395	9,309
M1. Two-factor CFA	82.595 (26)*	0.962	0.948	0.075	[0.057; 0.094]	8,923	9,062	9,034	8,945
M2. Bifactor CFA	39.379 (18)*	0.986	0.971	0.056	[0.032; 0.079]	8,871	9,050	9,014	8,899
M3. M1 with outcomes	801.100 (436)*	0.935	0.926	0.047	[0.042; 0.052]	34,947	35,561	35,437	35,043
M4. M2 with outcomes	745.480 (422)*	0.942	0.932	0.045	[0.039; 0.050]	34,902	35,585	35,447	35,009
**Study 2**									
M0. One-factor model	1,266.738 (27)*	0.735	0.647	0.208	[0.199; 0.218]	28,133	28,294	28,267	28,181
M1. Two-factor CFA	212.089 (26)*	0.960	0.945	0.082	[0.072; 0.093]	26,270	26,437	26,409	26,320
M2. Bifactor CFA	132.070 (18)*	0.976	0.951	0.077	[0.065; 0.090]	26,116	26,331	26,295	26,181
M3. M1 with covariates	917.084 (242)*	0.959	0.953	0.051	[0.048; 0.055]	72,705	73,194	73,112	72,851
M4. M2 with covariates	770.468 (231)*	0.967	0.960	0.047	[0.043; 0.051]	72,512	73,067	72,974	72,678

CFA, confirmatory factor analyses; χ^2^, scaled χ^2^ test of exact fit; df, degrees of freedom; CFI, comparative fit index; TLI, Tucker-Lewis index; RMSEA, root mean square error of approximation; 90% CI, 90% confidence interval. *p < 0.05.

Parameter estimates from both of these models estimated in both samples are reported in Table 2 and were well-aligned with those initial observations. Indeed, whereas the two-factor CFA model resulted in well-defined interpersonal (Sample 1: λ = 0.752 to 0.957; ω = 0.926; Sample 2: 0.541 to 0.948; ω = 0.897) and informational (Sample 1: λ = 0.759 to 0.906; ω = 0.917; Sample 2: λ = 0.719 to 0.858; ω = 0.887) justice factors, the latent correlation between these factors (Sample 1: *r* = 0.754; Sample 2: *r* = 0.732) was high enough to suggest that their simultaneous inclusion in predictive analyses was likely to result in multicollinearity due to partial conceptual overlap. This partial overlap was captured very well in the bifactor CFA model through the estimation of a well-defined G factor reflecting participants’ global perceptions of interactional justice (Sample 1: λ = 0.610 to 0.861; ω = 0.953; Sample 2: λ = 0.479 to 0.785; ω = 0.937). Still, this bifactor representation also succeeded in capturing the unique nature of an interpersonal justice S factor (Sample 1: λ = 0.440 to 0.594; ω = 0.824; Sample 2: λ = 0.263 to 0.599; ω = 0.771) and of a slightly weaker informational justice S factor (Sample 1: λ = -0.141 to 0.466; ω = 0.605; Sample 2: λ = -0.071 to 0.657; ω = 0.495). The weaker factor loadings associated with the informational justice factor suggested that more limited specificity remained in these indicators once the variance explained by global perceptions of interactional justice was considered. It is typical for bifactor models to result in S factors that are not defined as strongly as in CFA and even in a subset of weakly defined S factors (Morin et al., 2016). The relative strength of these S factors simply reflects the relative amount of global, relative to specific, variance included in the items forming these subscales.

**TABLE 2:** Standardized factor loadings (λ) and uniquenesses (δ) for the measurement models (Study 1).

	Two-factor CFA (Sample 1)		B-CFA (Sample 1)			Two-factor CFA (Sample 2)			B-CFA (Sample 2)	
Items	λ	δ	G λ	S λ	δ	λ	δ	G λ	S λ	δ
**Interpersonal justice**										
Item 1	0.831	0.309	0.632	0.546	0.302	0.844	0.287	0.627	0.570	0.282
Item 2	0.957	0.085	0.752	0.594	0.082	0.932	0.131	0.716	0.599	0.128
Item 3	0.928	0.138	0.749	0.544	0.142	0.948	0.102	0.740	0.590	0.105
Item 4	0.752	0.435	0.610	0.440	0.435	0.541	0.707	0.479	0.263	0.701
ω	0.926			0.824		0.897			0.771	
**Informational justice**										
Item 1	0.759	0.423	0.852	*−0.141*	0.255	0.731	0.465	0.779	*−0*.*026*	0.393
Item 2	0.821	0.326	0.782	0.221	0.339	0.832	0.308	0.740	0.657	0.021
Item 3	0.906	0.179	0.861	0.232	0.204	0.858	0.264	0.785	*0.286*	0.303
Item 4	0.859	0.263	0.781	0.466	0.173	0.765	0.415	0.729	*0.190*	0.432
Item 5	0.795	0.368	0.731	0.356	0.339	0.719	0.483	0.776	*−0*.*071*	0.393
ω	0.917		0.953	0.605		0.887		0.937	0.495	

*CFA, confirmatory factor analyses; B-CFA, bifactor confirmatory factor analyses; G, global factor estimated as part of a bifactor model; S, specific factor estimated as part of a bifactor model; λ, factor loading; δ, item uniqueness; ω, omega coefficient of model-based composite reliability. Non-significant parameters (p ≥ 0.05) are marked in italics.*

For comparative purposes, both models were retained for the predictive analyses, and the predictive models including the outcome variables resulted in an acceptable level of model fit (Table 1). The two-factor CFA model suggested that perceptions of interpersonal justice were negatively related to cognitive weariness, physical fatigue, negative affect and positively related to affective commitment, normative commitment, and altruism (Table 3). Employees’ informational justice perceptions were positively related to their affective and normative commitment to their organization. In contrast, for the bifactor CFA model, most of these relations were mainly related to employees’ global levels of interactional justice perceptions, with no remaining effects associated with their specific perceptions of interpersonal and interactional justice. The relations between the G factor and employees’ levels of life satisfaction, physical fatigue, cognitive weariness, affective commitment, and normative commitment were stronger in magnitude than those identified in the two-factor CFA model. The bifactor CFA models revealed additional positive relations between global interactional justice perceptions and levels of positive affect, or continuance commitment (high sacrifice), and civic virtue. However, the relation between the global interactional justice factor and negative affect was not statistically significant in bifactor CFA, although it was of a comparable magnitude and direction to the similar relation identified in the two-factor CFA model, and much higher than the relation between the specific interpersonal factor and the same outcome in the bifactor CFA solution. This apparent change thus simply appeared to be related to the slightly lower precision of that estimate in the bifactor CFA model (with a SE of 0.127 relative to 0.075), possibly due to greater model complexity. This result suggested that once global levels of interactional justice perceptions were considered the direction of relations between justice perceptions and affect was positive and related to positive (rather than negative) affect. Finally, no relation between justice perceptions, emotional exhaustion, and the low alternative component of continuance commitment could be identified in any of these models.

**TABLE 3:** Effects of organizational justice on outcomes (Study 1).

	Negative affect β (SE)	Positive affect β (SE)	Life satisfaction β (SE)	Physical fatigue β (SE)	Cognitive weariness β (SE)	Emotional exhaustion β (SE)
**Two-factor CFA**						
Interpersonal	−0.237(0.075)**	0.129 (0.089)	0.312(0.081)**	−0.178(0.072)*	−0.215(0.072)**	−0.151(0.083)
Informational	0.031 (0.080)	0.024 (0.088)	−0.002(0.077)	−0.118(0.068)	−0.027(0.068)	−0.037(0.079)
**Bifactor CFA**						
S: Interpersonal	−0.078(0.304)	−0.116(0.260)	−0.136(0.246)	0.282 (0.311)	−0.003(0.158)	−0.212(0.184)
S: Informational	0.017 (0.149)	−0.072(0.146)	−0.142(0.253)	0.129 (0.332)	0.028 (0.112)	−0.109(0.148)
G: Interactional	−0.198(0.127)	0.203(0.102)*	0.389(0.147)**	−0.401(0.200)*	−0.254(0.078)**	−0.109(0.108)

	**Affective commitment β (SE)**	**Normative commitment β (SE)**	**High sacrifice commitment β (SE)**	**Low alternatives commitment β (SE)**	**Altruism β (SE)**	**Civic virtue β (SE)**

**Two-factor CFA**						
Interpersonal	0.271(0.080)**	0.173(0.076)*	0.157 (0.097)	−0.005(0.106)	0.254(0.086)**	0.083 (0.095)
Informational	0.188(0.088)*	0.205(0.084)*	0.175 (0.104)	−0.106(0.103)	−0.022(0.082)	0.094 (0.097)
**Bifactor CFA**						
S: Interpersonal	0.125 (0.065)	0.072 (0.065)	0.057 (0.076)	−0.013(0.079)	0.145 (0.067)	0.008 (0.075)
S: Informational	−0.066(0.049)	−0.038(0.046)	−0.063(0.064)	−0.005(0.100)	−0.026(0.068)	−0.102(0.063)
G: Interactional	0.428(0.054)**	0.360(0.054)**	0.323(0.061)**	−0.097(0.067)	0.188(0.066)**	0.192(0.060)**

CFA, confirmatory factor analyses; G, global factor estimated as part of a bifactor model; S, specific factor estimated as part of a bifactor model; β, standardized estimate; SE, standard error of the coefficient. *p < 0.05. **p < 0.01.

### Discussion

A comparison between first-order and bifactor solutions supported the superiority of the bifactor CFA model: a better-fitting model coupled with a well-defined G factor representing employees’ global interactional justice perceptions coexisting with S factors reflecting the unique facets of their interpersonal and interactional justice perceptions left unexplained by the G factor. Whereas results obtained from the first-order model mainly suggested that the relations between justice perceptions and the outcomes variables mainly involved the interpersonal justice component, results from the more accurate bifactor CFA models rather showed these effects to be entirely explained by employees’ global interactional justice perceptions. More precisely, and in accordance with prior research (Ambrose et al., 2013; Buengeler and Den Hartog, 2015), our findings revealed that workers’ global perceptions of interactional justice were significantly associated with nine (positive affect, life satisfaction, physical fatigue, cognitive weariness, affective commitment, normative commitment, continuance commitment-high sacrifice, altruism, and civic virtue) of twelve (negative affect, emotional exhaustion, and continuance commitment-low alternatives) outcomes considered in this study. In particular, and attesting to the added value of this bifactor representation, the negative effects of global interactional justice perceptions on physical fatigue and cognitive weariness were even stronger in magnitude than those involving interpersonal justice in the two-factor CFA model. Likewise, the positive effects of global interactional justice perceptions on positive affect, life satisfaction, affective commitment, normative commitment, continuance commitment-high sacrifice, and civic virtue were also stronger in magnitude than those identified in the two-factor CFA model. However, the specific interpersonal and informational justice factors have no significant effect on the outcomes once employees’ global levels of interactional justice were considered. These results indicate that these S factors might not include a sufficient level of residual specificity to result in significant relations with the outcomes considered in this study, thus calling into question the need to consider these components once participants’ global interactional justice perceptions are considered. In the next study, we further verify this assertion by relying on LPA to examine the combined effects of all three components of interactional justice.

## Study 2

From the extensive examination of the dimensionality and outcomes of employees’ ratings of interactional justice conducted in Study 1, we seek to identify employees’ profiles of global and specific interactional justice perceptions in Study 2. Lacking guidance from previous person-centered studies relying on global and specific levels of interactional justice, we leave as an open research question the expected nature of the profiles that will be identified. However, it was expected that a relatively small number of profiles (i.e., between three and five) would be identified.

We also extend the results from Study 1 by examining the effects of employees’ perceptions of their supervisors’ transformational leadership practices on profile membership. Transformational leaders “broaden and elevate the interests of their employees, (…) generate awareness and acceptance of the purposes and mission of the group, and (…) stir employees to look beyond their own self-interest for the good of the group” (Bass, 1990, p. 21). Past studies examined the relationships between transformational leadership and outcomes of interest (e.g., job satisfaction, organizational commitment, and job performance; Munir et al., 2012). The links between transformational leadership and interactional justice are also well-documented (e.g., Wu et al., 2007). More specifically, Carter et al. (2014) showed that transformational leadership positively predicted interactional justice. Indeed, van Knippenberg et al. (2007) showed that interactional justice is a consequence of leadership. Moreover, because transformational leaders show appreciation and concern for employees (Bass, 1990), they may prime workers’ feelings of being treated with dignity, respect, and equality (i.e., interactional justice). Despite the well-documented importance of transformational leadership in the work context (Brown and May, 2012), no person-centered research has yet examined the effects of transformational leadership on interactional justice perception profiles. However, results from prior variable-centered studies (Carter et al., 2014) suggest that transformational leadership should be an important predictor of membership into profiles with high levels of interactional justice.

Finally, we also consider two ill-being indicators (anxiety and emotional exhaustion) as possible outcomes of the profiles. Interactional justice is considered as a psychological resource for workers (Gillet et al., 2015). Past variable-centered studies have thus, not surprisingly, demonstrated that interactional justice was associated with various positive outcomes (e.g., lower exhaustion and anxiety; Semmer et al., 2015). However, lacking guidance from previous person-centered studies, precise hypotheses cannot be formulated. In accordance with prior studies (Choi, 2008; Loi et al., 2009) showing that the effects of distinct justice components tended to be maximized when levels of the other justice components are low, we nevertheless expect that the most adaptive outcomes will be associated with profiles characterized by high scores on one specific dimension (e.g., interpersonal justice) and low scores on the other one (e.g., informational justice).

### Methods

#### Participants and Procedures

This study relied on data collection procedures identical to those used in Study 1, resulting in a sample of 1,057 workers (441 men, 616 women) from various organizations (e.g., public hospitals, industries, sales, and services) located in France. Respondents were aged between 18 and 62 years [mean, 42.71 (SD, 9.59) years], had an average organizational tenure of 12.50 (SD, 9.46) years, and an average tenure in the current position of 7.16 (SD, 6.86) years. A total of 942 participants were full-time workers (89.1%), whereas 958 participants were permanent workers (90.6%), and 99 were temporary workers (9.4%). Fifty-three participants (5.0%) had no diploma, 219 had a vocational training certificate (20.7%), 170 had a high school diploma (16.1%), and 615 had a university diploma (58.2%).

#### Measures

Participants’ perceptions of interpersonal justice (α = 0.92) and informational justice (α = 0.93), as well as their levels of emotional exhaustion (α = 0.87), were assessed as in Study 1.

##### Transformational leadership

Seven items (α = 0.96; e.g., “My manager gives encouragement and recognition to staff”) from Carless et al. (2000) were used to assess employees’ perceptions of transformational leadership from their supervisor. Items were rated on a seven-point Likert scale (1 = totally disagree to 7 = completely agree).

##### Anxiety

The Job-Anxiety Scale (Linden et al., 2008) was used to assess anxiety (five items; α = 0.86; e.g., “Colleagues or family have already told me that I am worrying too much about my work”). Items were rated on a seven-point Likert scale (1 = totally disagree to 7 = totally agree).

### Analyses

#### Preliminary Analyses

Preliminary analyses were first conducted, following the analytical strategy adopted in Study 1, to verify whether employees’ justice perceptions would follow the same structure as in this study. As in Study 1, the MLR estimator with FIML procedure was used to handle missing responses at the item level (0.00%–1.04%). Both of the *a priori* models (two-factor CFA and bifactor CFA) achieved an acceptable level of model fit, whereas the one-factor model failed to do so (Table 1). Furthermore, these analyses again supported the superiority of the bifactor CFA model relative to the two-factor CFA model (ΔCFI = +0.016; and lower values on all information criteria). Parameter estimates from both models are reported in Supplementary Table S1 and were well-aligned with those obtained in Study 1. To assess the similarity of these solutions across studies, we conducted sequential tests of measurement invariance (Millsap, 2011): (a) configural invariance, (b) weak invariance (loadings), (c) strong invariance (loadings, intercepts), (d) strict invariance (loadings, intercepts, uniquenesses), (e) invariance of the latent variances–covariances (loadings, intercepts, uniquenesses, variances–covariances), and (f) latent means invariance (loadings, intercepts, uniquenesses, variances–covariances, latent means). The results from these tests are reported in Supplementary Table S2 and support the comparability of these solutions across samples.

Factor scores were saved from the retained bifactor CFA solution and used as inputs for the person-centered analyses (Morin et al., 2017). To obtain factors scores on the predictor and outcome variables, CFA factors representing these covariables (transformational leadership, anxiety, and emotional exhaustion) were integrated to these measurement models and simply specified as correlated with the justice factors.

#### Person-Centered Analyses

Latent profile analysis solutions including one to eight profiles were estimated with the MLR estimator using the bifactor CFA factor scores as profile indicators. We relied on LPA models in which the means of the indicators were freely estimated across profiles. To avoid local maximum, we relied on 5,000 random sets of start values and 1,000 iterations and retained the 200 best solutions for final stage optimization (Hipp and Bauer, 2006). The procedure used to determine the optimal number of profiles, as well as alternative results based on factor scores taken from the two-factor CFA solution, is reported in the Supplementary Material. Once the optimal number of profiles has been selected, multinomial logistic regressions were conducted to test the relations between employees’ perceptions of transformational leadership and their likelihood of membership into the various profiles. Outcome levels were contrasted using Lanza et al’s. (2013) model-based approach implemented in Mplus through the Auxiliary (DCON) function (Asparouhov and Muthén, 2014).

### Results

#### Interactional Justice Profiles

**FIGURE 1:**
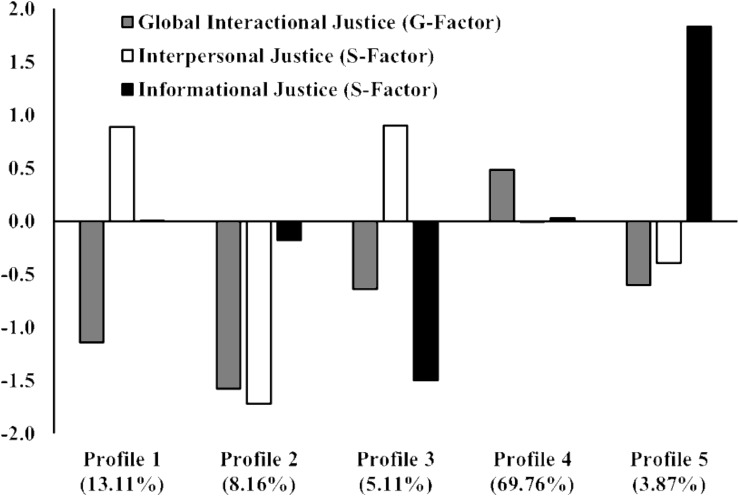
Final five-profile solution (bifactor CFA factor scores). Profile 1: high interpersonal/average informational; Profile 2: low interpersonal; Profile 3: high interpersonal/low informational; Profile 4: normative; Profile 5: high informational.

The results from the LPA models based on bifactor CFA factor scores converged on a five-profile solution (the rationale for this decision is provided in the Supplementary Material). This solution is illustrated in Figure 1; the exact within-profile means are reported in Supplementary Table S4, and the classification accuracy of participants into their most likely profile is reported in Supplementary Table S5. These results indicate a high classification accuracy, varying from 80.4 to 93.9%. We identified a *normative* profile (Profile 4), representing 69.76% of the employees, with satisfactory levels of global perceptions of interactional justice (approximately 0.5 SD higher than the sample average) and average levels of specific perceptions of interpersonal or informational justice. In contrast, all remaining profiles were characterized by moderately low (Profiles 3 and 5) to very low (Profiles 1 and 2) global perceptions of interactional justice, coupled with more differentiated perceptions of interpersonal or informational justice.

Thus, members of Profile 1 were characterized by average levels of justice perceptions specific to the informational area and by high levels of justice perceptions specific to the interpersonal area. This *high interpersonal/average informational* profile characterized a relatively large percentage (13.11%) of the remaining employees. In contrast, members of Profile 2 displayed very low justice perceptions specific to the interpersonal area, while also displaying average levels of justice perceptions specific to the informational area. This *low interpersonal* profile characterized 8.16% of the employees. Like Profile 1, Profile 3 characterized employees presenting high levels of justice perceptions specific to the interpersonal area. However, these employees also displayed very low justice perceptions specific to the informational area. This *high interpersonal*/*low informational* profile characterized 5.11% of the employees. Finally, members of Profile 5 were characterized by very high levels of justice perceptions specific to the informational area and moderately low levels of justice perceptions specific to the interpersonal area. This *high informational* profile characterized 3.87% of the employees.

#### Predictors of Profile Membership

Employees’ perceptions of transformational leadership have a significant effect on profile membership (Table 4). More precisely, higher perceptions of transformational leadership predicted a higher likelihood of membership into the *normative* (Profile 4) profile relative to all other profiles. In addition, among the profiles characterized by lower global levels of interactional justice perceptions (Profiles 1, 2, 3, 5), higher perceptions of transformational leadership also predicted a higher likelihood of membership into the *high interpersonal*/*low informational* (Profile 3) profile relative to Profiles 1, 2, and 5, into the *high informational* (Profile 5) profile relative to Profiles 1 and 2, and into the *high interpersonal/average informational* (Profile 1) profile relative to the *low interpersonal* (Profile 2) one. Interestingly, some of these comparisons could reflect the presence of a globally higher level of global interactional justice perceptions in the *high interpersonal*/*low informational* and *high informational* profiles (Profiles 3, 5) relative to the *high interpersonal/average informational* and *low interpersonal* ones (Profiles 1, 2). However, they also show that transformational leadership perceptions were able to differentiate among profiles similar to one another in terms of global interactional justice perceptions by favoring the profiles characterized by higher levels of specific interpersonal justice perceptions.

**TABLE 4:** Results from multinomial logistic regressions for the effects of transformational leadership on profile membership (bifactor CFA).

	Profile 1 vs. profile 5		Profile 2 vs. profile 5		Profile 3 vs. profile 5		Profile 4 vs. profile 5		Profile 1 vs. profile 4	
	Coef. (SE)	OR	Coef. (SE)	OR	Coef. (SE)	OR	Coef. (SE)	OR	Coef. (SE)	OR
Leadership	−0.795 (0.326)*	0.452	−2.217 (0.551)***	0.109	1.370 (0.482)**	3.934	3.459 (0.388)***	31.794	−4.254 (0.363)***	0.014

	**Profile 2 vs. profile 4**		**Profile 3 vs. profile 4**		**Profile 1 vs. profile 3**		**Profile 2 vs. profile 3**		**Profile 1 vs. profile 2**	
	**Coef. (SE)**	**OR**	**Coef. (SE)**	**OR**	**Coef. (SE)**	**OR**	**Coef. (SE)**	**OR**	**Coef. (SE)**	**OR**

Leadership	−5.676 (0.641)***	0.003	−2.090 (0.352)***	0.124	−2.164 (0.473)***	0.115	−3.586 (0.706)***	0.028	1.422 (0.429)**	4.145

SE, standard error of the coefficient; OR, odds ratio. The coefficients and OR reflects the effects of the predictor on the likelihood of membership into the first listed profile relative to the second listed profile; Transformational leadership is estimated from factor scores with a mean of 0 and a standard deviation of 1. Profile 1: high interpersonal/average informational; Profile 2: low interpersonal; Profile 3: high interpersonal/low informational; Profile 4: normative; Profile 5: high informational. *p < 0.05. **p < 0.01. ***p < 0.001.

#### Outcomes of Profile Membership

Multiple statistically significant differences emerged when outcome levels were compared across profiles (Table 5). In terms of anxiety, the lowest levels were observed within the *normative* profile (4), followed equally by *high interpersonal/average informational* (1), *high interpersonal*/*low informational* (3), *high informational* (5) profiles (although the levels of anxiety did not differ statistically from one another between Profiles 4 and 3), and finally by the *low interpersonal* (2) profile, which presented the highest levels of anxiety. In terms of emotional exhaustion, the lowest levels were equally observed in the *normative* (4) and *high informational* (5) profiles, followed by the remaining profiles. Among these other profiles, the only additional statistically significant difference reflected the fact that the *high interpersonal/average informational* (1) was characterized by lower levels of emotional exhaustion than the *low interpersonal* (2) one.

**TABLE 5:** Associations between profile membership and the outcomes (bifactor CFA).

	Profile 1 *M* [CI]	Profile 2 *M* [CI]	Profile 3 *M* [CI]	Profile 4 *M* [CI]	Profile 5 *M* [CI]	Significant differences
Anxiety	0.138 [−0.027; 0.303]	0.870 [0.635; 1.105]	0.037 [−0.212; 0.286]	−0.144 [−0.205; −0.083]	0.208 [−0.102; 0.518]	2 > 1 = 3 = 5; 3 = 4; 2 > 4; 1 = 5 > 4
Emotional exhaustion	0.125 [−0.038; 0.288]	0.413 [0.188; 0.638]	0.320 [0.042; 0.598]	−0.083 [−0.150; −0.016]	−0.239 [−0.506; 0.028]	2 = 3 > 4 = 5; 1 = 3; 2 > 1 > 4 = 5

*M, Mean; CI, 95% confidence interval. Indicators of anxiety and emotional exhaustion are estimated from factor scores with a mean of 0 and a standard deviation of 1. Profile 1: high interpersonal/average informational; Profile 2: low interpersonal; Profile 3: high interpersonal/low informational; Profile 4: normative; Profile 5: high informational.*

### Discussion

Replicating Study 1’s results, preliminary analyses supported a bifactor representation of interactional justice perceptions and even showed this representation to be fully invariant across the two studies. In addition, and supporting Morin et al. (2017) assertion that failure to control for coexisting global and specific constructs within a set of profile indicators would result in the estimation of *level*-differentiated profiles, alternative profiles estimated based on two-factor CFA factor scores (reported in the Supplementary Material) differed from one another only quantitatively (i.e., presenting very low, moderately low, low, moderately high, or high levels of justice across indicators). In contrast, when properly estimated from bifactor factor scores, our results revealed a set of five profiles presenting clear qualitative differences. A first profile characterized a majority of employees (69.76%) with moderately high global perceptions of interactional justice coupled with average specific perceptions of interpersonal and informational justice. This *normative* profile characterized a majority of employees for whom justice expectations were globally met (i.e., to match some desirable “normative” level) across dimensions. This profile was also associated with the lowest levels of anxiety and emotional exhaustion. In line with Study 1, these results confirm that global interactional justice was positively related to adaptive work outcomes (Ambrose et al., 2013; Buengeler and Den Hartog, 2015).

The remaining profiles were all characterized by moderately low to very low global perceptions of interactional justice, coupled with discrepant justice perceptions across the specific informational and interpersonal components. For these profiles, specific perceptions of interpersonal or informational justice mattered more. These profiles presented lower levels of global interactional justice perceptions coupled with (a) *high interpersonal/average informational* justice perceptions; (b) *low interpersonal* justice perceptions; (c) *high interpersonal*/*low informational* justice perceptions; and (d) *high informational* justice perceptions. When these four profiles were considered from an outcomes’ perspective, the *low interpersonal* profile (also characterized by the lowest levels of global interactional justice perceptions) appeared to present the highest levels of anxiety and emotional exhaustion, whereas lower levels of emotional exhaustion were also associated with the *high informational* profile. Finally, and in accordance with our expectations (Carter et al., 2014), employees’ perceptions of their manager’s transformational leadership behaviors tended to predict a higher likelihood of membership into profiles characterized by higher levels of global interactional justice, as well as into those characterized by higher specific levels of interpersonal justice perceptions.

## General Discussion

The present research adopts an approach advocated by Morin et al. (2017) to more specifically analyze the value of jointly considering global and specific dimensions of the interactional justice construct. Through the application of this framework, we were able to achieve an improved representation of the structure of employees’ interactional justice perceptions and to show how this representation provided a way to identify more clearly differentiated interactional justice profiles.

### Coexisting Global and Specific Components of Interactional Justice

In order to assess the presence of coexisting global and specific constructs, Morin and colleagues (Morin, 2016; Morin et al., 2017) propose to rely on bifactor measurement models. In the present research, the comparison of bifactor models with alternative first-order and one-factor models very clearly supported the superiority of a bifactor representation of interactional justice perceptions across three independent samples of employees. This bifactor solution revealed well-defined coexisting factors representing employees’ global interactional justice perceptions and more specific interpersonal and informational justice perceptions. Interactional justice perceptions might thus reflect a global overarching factor (Ambrose et al., 2013; Buengeler and Den Hartog, 2015), and this G factor may coexist with S factors reflecting workers’ perceptions of interpersonal and informational justice remaining unexplained by the G factor (Kass, 2008). More generally, bifactor models allowed us to disaggregate the effects attributable to global interactional justice perceptions in comparison to the two more specific interactional justice components (interpersonal and informational justice). This improved representation of interactional justice perceptions represents a major contribution of the current research.

From an outcomes’ perspective, the present results supported our expectations (e.g., Gillet et al., 2015; Wang and Jiang, 2015) in demonstrating the key role of employees’ global interactional justice perceptions in the prediction of a variety of individually relevant (positive affect, life satisfaction, physical fatigue, and cognitive weariness) and organizationally relevant (affective commitment, normative commitment, continuance commitment: high sacrifice, altruism, and civic virtue) outcomes. However, they also went against our expectations (e.g., Gupta and Kumar, 2013) in failing to demonstrate the added value of specific perceptions of informational and interpersonal justice in the prediction of any of the outcome variables. Although these variable-centered results call into question the need to consider these components once participants’ global interactional justice perceptions are considered, we decided to more thoroughly verify this assertion via the adoption of a person-centered configurational approach in Study 2.

### Interactional Justice Profiles

The reliance on a person-centered approach built from factor scores obtained as part of preliminary analyses allowed us to directly assess the combination of global and specific (interpersonal and informational justice) interactional justice components among different profiles of workers (Morin et al., 2017). We identified a large (69.8%) *normative* profile suggesting that, for the majority of the sample, global perceptions of interactional justice remain satisfactory and balanced with the specific perceptions of informational and interpersonal justice. Apart from this *normative* profile, all other profiles were characterized by lower global perceptions of interactional justice and by discrepant levels of interpersonal justice relative to informational justice perceptions. This last observation suggests that balanced perceptions are important to the *normative* profile. This result thus suggests that employees’ perceptions regarding interactional justice levels will be met as long as some perceptive threshold is met across both justice components. When these thresholds are met, moderate versus higher justice perceptions no longer matter.

Furthermore, lower levels of emotional exhaustion and anxiety were consistently associated with the *normative* profile. Consistent with past studies (Rego and Pina e Cunha, 2010; Ambrose et al., 2013), high levels of global interactional justice perceptions anchored in balanced levels of informational and interpersonal justice perceptions might thus reflect a combination leading to positive outcomes. The sheer size of this profile (close to 70%) might also explain why Study 1 failed to find predictive relations between specific perceptions of interpersonal and informational justice once global interactional justice perceptions were considered. Indeed, additional results suggest that these specific perceptions may indeed play a role.

One might wonder about the non-significant difference in emotional exhaustion levels that was observed between the *normative* profile and the *high informational* profile, characterized by moderately low levels of global interactional justice but very high levels of specific informational justice. Interestingly, employees corresponding to the *high informational* profile tended to present much more positive specific informational justice perceptions than those corresponding to the *Normative* profile. Not only is this observation consistent with the theoretically important role ascribed to informational justice (Au and Leung, 2016), but it also suggests that high levels of informational justice may help employees to achieve desirable outcomes even in the presence of moderately low global levels of interactional justice perceptions. However, this compensatory effect might be limited to emotional exhaustion without generalizing to anxiety. Future investigations should consider other positive (e.g., organizational commitment, organizational citizenship behaviors) and negative (e.g., workaholism, work–family conflict) consequences.

The *low interpersonal* profile was identified as the least desirable. Yet, this profile was characterized by very low levels of global interactional justice perceptions and average levels of specific informational justice perceptions that matched those observed in the *high interpersonal/average informational* profile. However, the *high interpersonal/average informational* profile was also characterized by high levels of specific interpersonal justice perceptions. Thus, whereas very high levels of informational justice perceptions helped to achieve positive outcomes in the presence of moderately low global interactional justice perceptions, high levels of interpersonal justice perceptions may play a similar role against the development of undesirable outcomes in the presence of low global interactional justice perceptions.

These interpretations were supported by finding that the *high interpersonal/low informational* profile fell in between the *low interpersonal* and the *high informational* profiles. Yet, whereas this *high interpersonal/low informational* profile is characterized by moderately low global levels of interactional justice perceptions matching the levels observed in the *high informational* profile, this profile is also characterized by high levels of specific interpersonal justice perceptions and very low levels of specific informational justice perceptions. Yet, this profile is also associated with worse outcomes (i.e., higher levels of emotional exhaustion) than that the *high informational* profile, which reinforces our interpretation that high specific perceptions of informational justice may help to compensate for moderately low levels of global interactional justice. Furthermore, this profile remains slightly more adaptive than the *low interpersonal* one (i.e., lower levels of anxiety), suggesting that higher levels of specific interpersonal justice perceptions may partly protect employees’ against the negative effects of low levels of global interactional justice perceptions. Taken together, these results are well-aligned with those from past research (e.g., Choi, 2008; Loi et al., 2009) showing that the effects of distinct justice components tended to be maximized when levels of the other justice components are low.

Study 2 also sought to address the lack of studies on the predictors of interactional justice profiles. We considered the possible role of transformational leadership and found that it was associated with an increased likelihood of membership into the profiles characterized by higher levels of global interactional justice perceptions (*normative* profile) and higher levels of specific interpersonal justice perceptions (*high interpersonal/low informational* profile). These findings are aligned with our expectations and past studies (Carter et al., 2014). However, additional studies are necessary to unpack the mechanisms underlying this association.

These profiles support the added value of considering global and specific levels of interactional justice. More generally, these findings confirm that, when considered from a configural perspective, specific interactional justice facets are differentially related to outcomes once global levels of interactional justice are taken into account. However, future research is necessary to assess the generalizability of these profiles while also taking into account workers’ perceptions of procedural and distributive justice.

### Limitations and Directions for Future Research

Although the present research offers the first investigation of the characteristics, determinants, and consequences of employees’ interactional justice profiles, it has some limitations. First, this study capitalized on self-report measures, which may have been influenced by self-reported biases and social desirability. Upcoming studies should incorporate more objective indicators of organizational and individual functioning (e.g., absenteeism), as well as ratings obtained from multiple informants (e.g., supervisors’ ratings of performance). Second, we examined covariates specified as predictors (transformational leadership) or outcomes (e.g., emotional exhaustion, organizational commitment) based on theoretical and empirical considerations (e.g., Colquitt, 2001). However, future longitudinal studies would gain from studying the direction of the relations between determinants, consequences, and profiles. In addition, longitudinal research would make it possible to test whether membership into these various profiles remains stable over time (Kam et al., 2016). Third, future research should consider other predictors of changes in interactional justice profiles (e.g., organizational culture, authentic leadership). Finally, we relied on convenience samples of French workers and were not able to consider the potential multilevel structure of the data and highlight the importance for future studies to rely on more diversified (e.g., professions, languages, cultures) and representative samples.

### Practical Implications

Managers should pay attention to workers with low global levels of interactional justice perceptions and especially to those who also display low levels of interpersonal justice as these workers have high levels of burnout and anxiety. Interestingly, findings from Study 2 showed that transformational leadership was associated with a lower likelihood of membership into this least desirable profile (*low interpersonal*). Furthermore, numerous investigations, as well as Study 2, showed that transformational leadership was positively related to interactional justice (e.g., Carter et al., 2014). Practitioners may thus work at building management training programs encouraging supervisors to build trustful relationships with their teams, to increase workers’ sense of the collective interests, and to help employees achieve collective goals (Brown and May, 2012). For instance, in a quasi-experimental study, Parry and Sinha (2005) showed that transformational leadership behaviors were more frequent among individuals who followed a training program focusing on the development and learning of a variety of leadership skills including self-planning, self-analysis, and coaching of leadership activities and refection upon experiences. Employees’ satisfaction with their leader and extra effort were also improved as a result of this training. More generally, organizations should develop leadership programs for their managers, resulting in increases in employees’ perceptions of interactional justice and positive outcomes (Gillet et al., 2016).

## Data Availability Statement

The datasets generated for this study are available on request to the corresponding author.

## Ethics Statement

Ethical review and approval was not required for the study on human participants in accordance with the local legislation and institutional requirements. The patients/participants provided their written informed consent to participate in this study.

## Author Contributions

EF, AM, and NG contributed to the conception and design of the study, performed the statistical analysis, and wrote the first draft of the manuscript. All authors organized the database, contributed to the manuscript revision, read, and approved the submitted version.

## Conflict of Interest

The authors declare that the research was conducted in the absence of any commercial or financial relationships that could be construed as a potential conflict of interest.
