# Supplementary material for: On the Value of Considering Specific Facets of Interactional Justice Perceptions
Source: Front Psychol. 2020 May 15;11:812. doi: 10.3389/fpsyg.2020.00812 (PMC7242617; doi:10.3389/fpsyg.2020.00812)
Supplement: Supplementary file 1 [file Data_Sheet_1.docx]

**Online Supplemental Materials for:**

**On the Value of Considering Specific Facets of Interactional Justice Perceptions**

**Determination of the Optimal Number of Profiles**

To determine the optimal number of profiles at both time points, multiple sources of information need to be considered, including the examination of the substantive meaningfulness, theoretical conformity, and statistical adequacy of the solutions (Marsh, Lüdtke, Trautwein, & Morin, 2009; Morin, 2016; Muthén, 2003). In addition, statistical indices are available to support this decision: (i) The Akaïke Information Criterion (AIC), (ii) the Consistent AIC (CAIC), (iii) the Bayesian Information Criterion (BIC), (iv) the sample-size Adjusted BIC (ABIC), (v) the standard and adjusted Lo, Mendell and Rubin’s (2001) Likelihood Ratio Tests (LMR/aLMR; as these tests typically yield the same conclusions, we only report the aLMR), and (vi) the Bootstrap Likelihood Ratio Test (BLRT). A lower value on the AIC, CAIC, BIC, and ABIC suggests a better-fitting model. The aLMR and BLRT compare a *k*‑class model with a *k-1*‑class model. A significant *p* value indicates that the *k-1*‑class model should be rejected in favor of a *k‑*class model. Simulation studies indicate that four of these indicators (CAIC, BIC, ABIC, and BLRT) are effective (e.g., Nylund, Asparouhov, & Muthén, 2007; Peugh & Fan, 2013; Tein, Coxe, & Cham, 2013; Tofighi & Enders, 2008) and that the AIC and LMR/ALMR should not be used in the class enumeration process as they respectively tend to over- and under-extract incorrect number of profiles (e.g., Diallo, Morin, & Lu, 2016, 2017; Nylund et al., 2007; Peugh & Fan, 2013; Tofighi & Enders, 2008). These indicators will thus be reported only to ensure a complete disclosure, but will not be used to select the optimal number of profiles. It should be noted that these tests remain heavily influenced by sample size (Marsh et al., 2009), so that with sufficiently large samples, they may keep on suggesting the addition of profiles without reaching a minimum. In this situation, the point at which these indicators appears to reach a plateau can be used to suggest the optimal solution (Morin et al., 2011). Finally, the entropy indicates the precision with which the cases are classified into the various profiles. The entropy should not be used to determine the optimal number of profiles (Lubke & Muthén, 2007), but summarizes classification accuracy (0 to 1).

The fit indices associated with the LPA estimated from both the two-factor CFA and bifactor-CFA factor scores are reported in Table S3 of these online supplements, and the elbow plots associated with these results are presented in Figures S1 and S2 of these online supplements. Examination of these results revealed that most indices kept on improving with the addition of profiles to the solution, without reaching a minimum, with the sole exception of the CAIC which supported the 7-profile solution for the models based on the bifactor-CFA factor scores. Turning our attention to the elbow plots, they suggested two separate points of inflexion for the models based on the bifactor-CFA factor scores, one located at 3 profiles and another located at 6 profiles. For models based on the two-factor CFA factor scores, the elbow plot similarly suggested two points of inflexion corresponding to the 3- and 7-profile solutions.

Given the clear superiority of the bifactor-CFA measurement models identified in both studies, we first turned our attention to the models based on bifactor-CFA factor scores. For the models based on bifactor-CFA factor scores and corresponding to the main results reported in the manuscript, we thus systematically compared solutions including 3 to 7 profiles. This examination showed that all solutions were fully proper statistically. Furthermore, this examination revealed that moving from a 3-profile solution to a 4-profile solution, and from a 4-profile solution to a 5-profile solution, resulted in the addition of meaningfully different profiles to the solution, whereas moving from a 5-profile solution to 6- or 7-profile solutions resulted in the arbitrary division of one profile into similar profiles. The 5-profile solution based on bifactor-CFA factor scores was thus retained for interpretation and for the next stages of analyses. For comparison purposes, the 5-profile solution based on two-factor CFA factor scores was also retained.

**Profiles Based on Two-Factor CFA Factor Scores**

The final LPA solution based on two-factor CFA factor scores is illustrated in Figure S3 of these online supplements, the exact within-profile means are reported in Table S4 of these online supplements, and the classification accuracy of participants into their most likely profile is reported in Table S5 of these online supplements. These results indicate a high classification accuracy, varying from 96.6% to 99.1% across profiles.

It is easy to see that the solution reflected what Morin and Marsh (2015) referred to as pure *level* (or quantitative) differences between profiles. More precisely, this solution revealed profiles characterized by matching levels on all of the indicators considered, which simply differed from one another quantitatively. Indeed, these profiles simply reflected very low (Profile 1), moderately low (Profile 2), low (Profile 3), high (Profile 4) or moderately high (Profile 5) levels of justice. Morin and Marsh (2015) noted that such a solution represent evidence against the added value of a person-centered solution, and similar results have also been previously interpreted as arguing against the added value of adopting a multidimensional representation of the construct under study (interactional justice in the present context) (e.g., Gillet, Morin, Cougot, & Gagné, 2017). However, Morin and colleagues (Morin et al., 2017; Morin & Marsh, 2015) also demonstrated that the observation of *level* differences could also be an artefact of the lack of proper disaggregation of the global versus specific components of the constructs under study. Based on these results and observations, coupled with the superiority of the bifactor-CFA representation of employee’s justice perceptions, this solution based on two-factor CFA factor scores will not be further considered in this study. However, interested readers can still consult results related to the integration of predictors and outcomes to this solution in Tables S6 and S7 of these online supplements. It is noteworthy that these additional results mainly support the ordering of the profiles based on the *level* differences described above, and could thus have been summarized by a simple multiple regression analysis relying on a single global score of justice perceptions.

**References Used in these Supplements**

Diallo, T.M.O, Morin, A.J.S., & Lu, H. (2016). Impact of misspecifications of the latent variance-covariance and residual matrices on the class enumeration accuracy of growth mixture models. *Structural Equation Modeling*, *23*, 507-531.

Diallo, T.M.O., **Morin, A.J.S.,** & Lu, H. (2017). The impact of total and partial inclusion or exclusion of active and inactive time invariant covariates in growth mixture. Psychological Methods, 22, 166-190.

Gillet, N., Morin, A.J.S., Cougot, B., & Gagné, M. (2017). Workaholism profiles: Associations with determinants, correlates, & outcomes. *Journal of Occupational & Organizational Psychology, 90*, 559-586.

Lo, Y., Mendell, N., & Rubin, D. (2001). Testing the number of components in a normal mixture. *Biometrika, 88*, 767-778.

Lubke, G., & Muthén, B.O. (2007). Performance of factor mixture models as a function of model size, covariate effects, and class-specific parameters. *Structural Equation Modeling, 14*, 26-47.

Marsh, H.W., Lüdtke, O., Trautwein, U., & Morin, A.J.S. (2009). Classical latent profile analysis of academic self-concept dimensions: Synergy of person- and variable-centered approaches to theoretical models of self-concept. *Structural Equation Modeling*, *16*, 191-225.

Morin, A.J.S. (2016). Person-centered research strategies in commitment research. In J.P. Meyer (Ed.), *The handbook of employee commitment* (p. 490-508). Cheltenham, UK: Edward Elgar.

**Morin, A.J.S.,** Boudrias, J.-S., Marsh, H.W., McInerney, D.M., Dagenais-Desmarais, V., Madore, I., & Litalien, D. (2017). Complementary variable- and person-centered approaches to exploring the dimensionality of psychometric constructs: Application to psychological wellbeing at work. Journal of Business and Psychology, 32, 395-419.

Morin, A.J.S., Maïano, C., Nagengast, B., Marsh, H.W., Morizot, J., & Janosz, M. (2011). Growth mixture modeling of adolescents trajectories of anxiety: The impact of untested invariance assumptions on substantive interpretations. *Structural Equation Modeling, 18*, 613-648.

Morin, A.J.S., & Marsh, H.W. (2015). Disentangling shape from levels effects in person-centered analyses: An illustration based on university teachers’ multidimensional profiles of effectiveness. *Structural Equation Modeling*, *22*, 39-59*.*

Muthén, B.O. (2003). Statistical and substantive checking in growth mixture modeling: Comment on Bauer and Curran (2003). *Psychological Methods, 8*, 369-377.

Nylund, K.L., Asparouhov, T., & Muthén, B.O. (2007). Deciding on the number of classes in latent class analysis and growth mixture modeling. *Structural Equation Modeling*, *14*, 535-569.

Peugh, J., & Fan, X. (2013). Modeling unobserved heterogeneity using latent profile analysis: A Monte Carlo simulation. *Structural Equation Modeling*, *20*, 616-639.

Tein, J.-Y., Coxe, S., & Cham, H. (2013). Statistical power to detect the correct number of classes in latent profile analysis. *Structural Equation Modeling*, *20*, 640-657.

Tofighi, D., & Enders, C. (2008). Identifying the correct number of classes in growth mixture models. In G.R. Hancock & K.M. Samuelsen (Eds.), *Advances in latent variable mixture models* (pp. 317-341). Charlotte, NC: Information Age.

**Table S1**

*Standardized Factor Loadings (λ) and Uniquenesses (δ) for the Measurement Models (Study 3)*

|  | Two-Factor CFA | | B-CFA | | |
| --- | --- | --- | --- | --- | --- |
| Items | λ | δ | G λ | S λ | δ |
| Interpersonal Justice |  |  |  |  |  |
| Item 1 | .852 | .275 | .690 | .501 | .273 |
| Item 2 | .974 | .052 | .808 | .549 | .046 |
| Item 3 | .966 | .067 | .818 | .510 | .071 |
| Item 4 | .717 | .485 | .669 | .286 | .472 |
| ω | .933 |  |  | .798 |  |
| Informational Justice |  |  |  |  |  |
| Item 1 | .810 | .344 | .842 | .142 | .271 |
| Item 2 | .912 | .169 | .772 | .512 | .143 |
| Item 3 | .903 | .185 | .757 | .520 | .156 |
| Item 4 | .848 | .281 | .746 | .384 | .296 |
| Item 5 | .793 | .371 | .755 | .245 | .370 |
| ω | .922 |  | .957 | .741 |  |

*Note*. CFA = Confirmatory factor analyses; G: Global factor estimated as part of a bifactor model; S: Specific factor estimated as part of a bifactor model; λ: Factor loading; δ: Item uniqueness; ω: Omega coefficient of model-based composite reliability.

**Table S2**

*Goodness-of-Fit Statistics for the Estimated Models: Tests of Measurement Invariance*

| Description | *χ*² (*df*) | CFI | TLI | RMSEA | 90% CI | AIC | CAIC | BIC | ABIC | CM | *∆χ²* (*df*) | ∆CFI | ∆TLI | ∆RMSEA |
| --- | --- | --- | --- | --- | --- | --- | --- | --- | --- | --- | --- | --- | --- | --- |
| Two-Factor CFA |  |  |  |  |  |  |  |  |  |  |  |  |  |  |
| M1. Configural invariance | 409.848 (78)* | .964 | .950 | .079 | [.072; .087] | 51060 | 51616 | 51532 | 51265 | - | - | - | - | - |
| M2. Weak invariance | 457.912 (92)* | .960 | .953 | .077 | [.070; .084] | 51083 | 51546 | 51476 | 51254 | M1 | 42.444 (14)* | -.004 | +.003 | -.002 |
| M3. Strong invariance | 613.876 (106)* | .945 | .944 | .084 | [.078; .091] | 51245 | 51616 | 51560 | 51382 | M2 | 188.742 (14)* | -.015 | -.009 | +.007 |
| M3’. Partial strong invariance | 523.109 (105)* | .955 | .953 | .077 | [.070; .083] | 51122 | 51499 | 51442 | 51261 | M2 | 68.518 (13)* | -.005 | .000 | .000 |
| M4. Strict invariance | 650.314 (123)* | .943 | .950 | .080 | [.074; .086] | 51349 | 51608 | 51569 | 51445 | M3’ | 114.799 (18)* | -.012 | -.003 | +.003 |
| M4’. Partial strict invariance | 597.145 (122)* | .949 | .954 | .076 | [.070; .082] | 51260 | 51524 | 51484 | 51357 | M3’ | 76.268 (17)* | -.006 | +.001 | -.001 |
| M5. Var-Cov invariance | 657.500 (128)* | .943 | .952 | .078 | [.072; .084] | 51333 | 51558 | 51524 | 51416 | M4’ | 64.560 (6)* | -.006 | -.002 | +.002 |
| M6. Latent means invariance | 919.533 (132)* | .915 | .930 | .094 | [.088; .100] | 51705 | 51904 | 51874 | 51778 | M5 | 366.337 (4)* | -.028 | -.022 | +.016 |
| M6’. Partial means invariance | 666.764 (130)* | .942 | .952 | .078 | [.072; .084] | 51339 | 51551 | 51519 | 51417 | M5 | 8.902 (2) | -.001 | .000 | .000 |
| Bifactor-CFA |  |  |  |  |  |  |  |  |  |  |  |  |  |  |
| M1. Configural invariance | 242.760 (54)* | .980 | .959 | .072 | [.063; .081] | 50810 | 51470 | 51416 | 51073 | - | - | - | - | - |
| M2. Weak invariance | 306.923 (84)* | .976 | .969 | .063 | [.055; .070] | 50848 | 51364 | 51286 | 51038 | M1 | 68.438 (30)* | -.004 | +.010 | -.009 |
| M3. Strong invariance | 331.564 (96)* | .974 | .971 | .060 | [.053; .067] | 50859 | 51295 | 51229 | 51020 | M2 | 25.194 (12) | -.002 | +.002 | -.003 |
| M4. Strict invariance | 482.629 (114)* | .960 | .962 | .069 | [.063; .075] | 51054 | 51372 | 51324 | 51172 | M3 | 135.032 (18)* | -.014 | -.009 | +.009 |
| M4’. Partial strict invariance | 413.025 (113)* | .967 | .969 | .063 | [.056; .069] | 50981 | 51305 | 51256 | 51101 | M3 | 74.565 (17)* | -.007 | -.002 | +.003 |
| M5. Var-Cov invariance | 476.111 (119)* | .961 | .965 | .067 | [.060; .073] | 51076 | 51361 | 51318 | 51181 | M4’ | 51.296 (6)* | -.006 | -.004 | +.004 |
| M6. Latent means invariance | 859.087 (125)* | .920 | .931 | .093 | [.087; .099] | 51606 | 51851 | 51814 | 51696 | M5 | 717.747 (6)* | -.041 | -.034 | +.026 |
| M6’. Partial means invariance | 488.550 (122)* | .960 | .965 | .067 | [.060; .073] | 51084 | 51349 | 51309 | 51182 | M5 | 12.592 (3)* | -.001 | .000 | .000 |

*Note:* * *p*< .05; *χ*²: Scaled chi-square test of exact fit; *df*: Degrees of freedom; CFI: Comparative fit index; TLI: Tucker-Lewis index; RMSEA: Root mean square error of approximation; 90% CI: 90% confidence interval; Var-Cov: Variance-covariance; CM: Comparison model; Δ: Change in fit relative to the CM.

**Table S3**

*Results from the Latent Profile Analysis Models*

| Model | LL | #fp | Scaling | AIC | CAIC | BIC | ABIC | Entropy | aLMR | BLRT |
| --- | --- | --- | --- | --- | --- | --- | --- | --- | --- | --- |
| *From Two-Factor CFA Factor Scores* |  |  |  |  |  |  |  |  |  |  |
| 1 Profile | -2956.988 | 4 | 1.111 | 5922 | 5946 | 5942 | 5929 | Na | Na | Na |
| 2 Profiles | -2543.720 | 7 | 1.343 | 5101 | 5143 | 5136 | 5114 | .885 | < .001 | < .001 |
| 3 Profiles | -2379.226 | 10 | 2.167 | 4778 | 4838 | 4828 | 4796 | .822 | .141 | < .001 |
| 4 Profiles | -2297.694 | 13 | 1.186 | 4621 | 4699 | 4686 | 4645 | .829 | < .001 | < .001 |
| 5 Profiles | -2136.561 | 16 | 1.772 | 4305 | 4400 | 4385 | 4334 | .966 | .080 | < .001 |
| 6 Profiles | -2014.420 | 19 | 1.636 | 4067 | 4180 | 4161 | 4101 | .975 | .025 | < .001 |
| 7 Profiles | -1892.729 | 22 | 1.297 | 3829 | 3961 | 3939 | 3869 | .983 | < .001 | < .001 |
| 8 Profiles | -1851.089 | 25 | 1.343 | 3752 | 3901 | 3876 | 3797 | .981 | .144 | < .001 |
| *From Bifactor-CFA Factor Scores* |  |  |  |  |  |  |  |  |  |  |
| 1 Profile | -4091.868 | 6 | 1.290 | 8196 | 8232 | 8226 | 8206 | Na | Na | Na |
| 2 Profiles | -3993.608 | 10 | 1.195 | 8007 | 8067 | 8057 | 8025 | .918 | < .001 | < .001 |
| 3 Profiles | -3878.435 | 14 | 1.356 | 7785 | 7868 | 7854 | 7810 | .827 | < .001 | < .001 |
| 4 Profiles | -3836.424 | 18 | 1.611 | 7709 | 7816 | 7798 | 7741 | .844 | .245 | < .001 |
| 5 Profiles | -3797.843 | 22 | 1.708 | 7640 | 7771 | 7749 | 7679 | .859 | .323 | < .001 |
| 6 Profiles | -3760.615 | 26 | 1.501 | 7573 | 7728 | 7702 | 7620 | .862 | .092 | < .001 |
| 7 Profiles | -3743.570 | 30 | 1.584 | 7547 | 7726 | 7696 | 7601 | .865 | .499 | < .001 |
| 8 Profiles | -3727.859 | 34 | 1.283 | 7524 | 7726 | 7692 | 7584 | .868 | .032 | < .001 |

*Note*: LL: Model LogLikelihood; #fp: Number of free parameters; Scaling: Scaling factor associated with MLR loglikelihood estimates; AIC: Akaïke Information Criteria; CAIC: Constant AIC; BIC: Bayesian Information Criteria; ABIC: Sample-size adjusted BIC; aLMR: Adjusted Lo-Mendel-Rubin likelihood ratio test; BLRT: Bootstrap Likelihood Ratio Test.


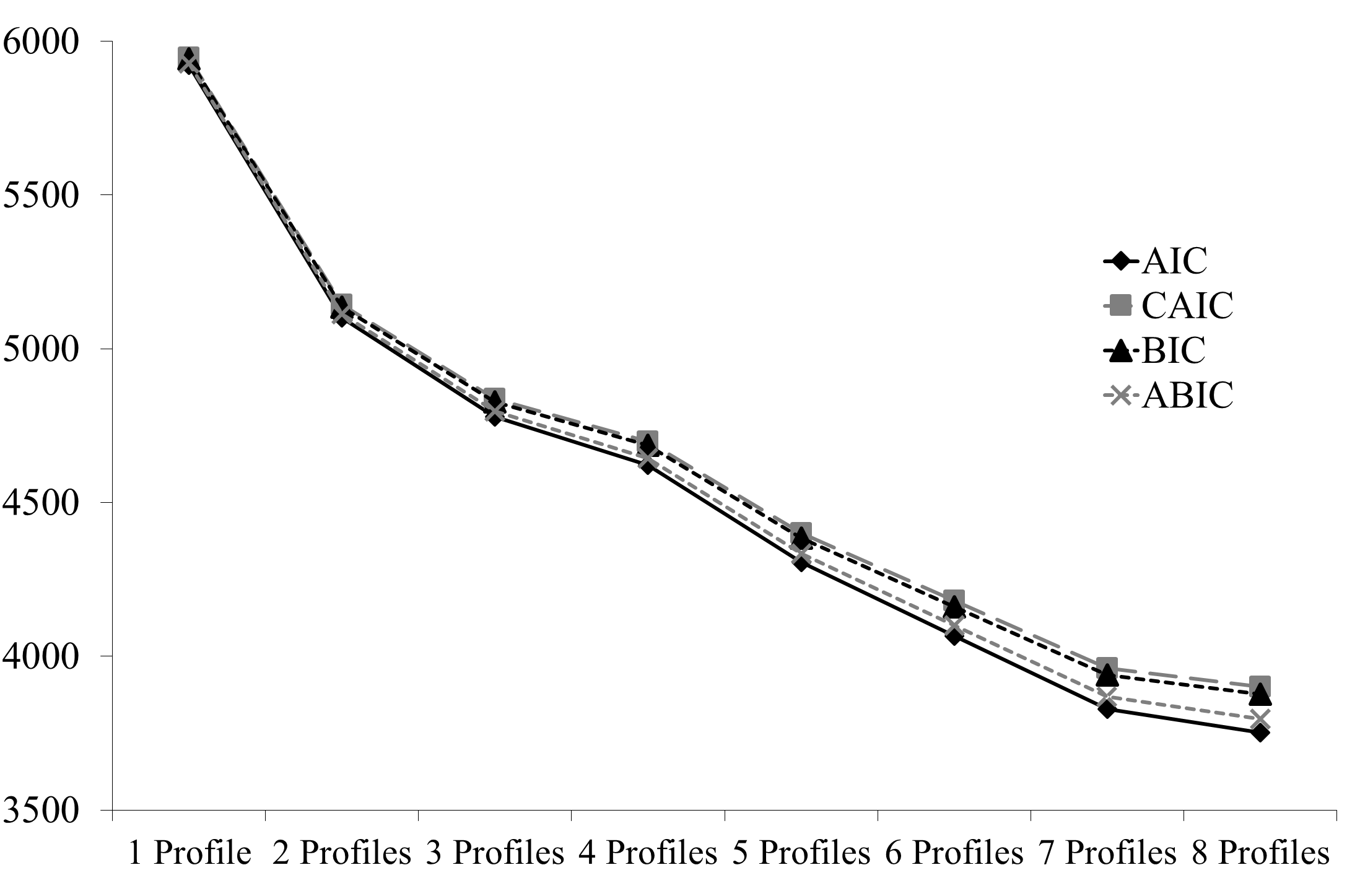


**Figure S1**

Elbow Plot of the Value of the Information Criteria for Solutions Including Different Number of Latent Profiles and Estimated from Two-Factor CFA Factor Scores


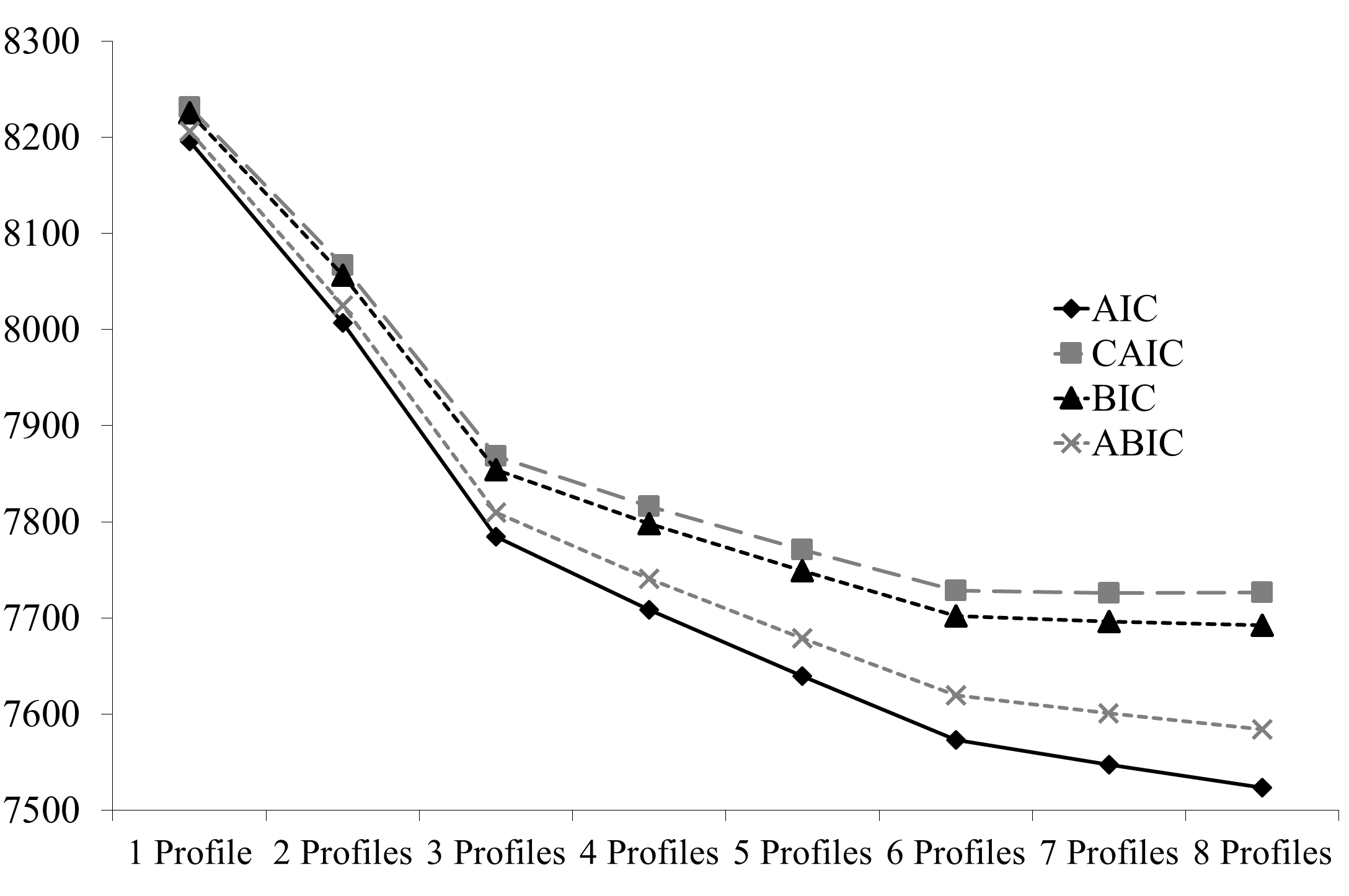


**Figure S2**

Elbow Plot of the Value of the Information Criteria for Solutions Including Different Number of Latent Profiles and Estimated from Bifactor-CFA Factor Scores

**
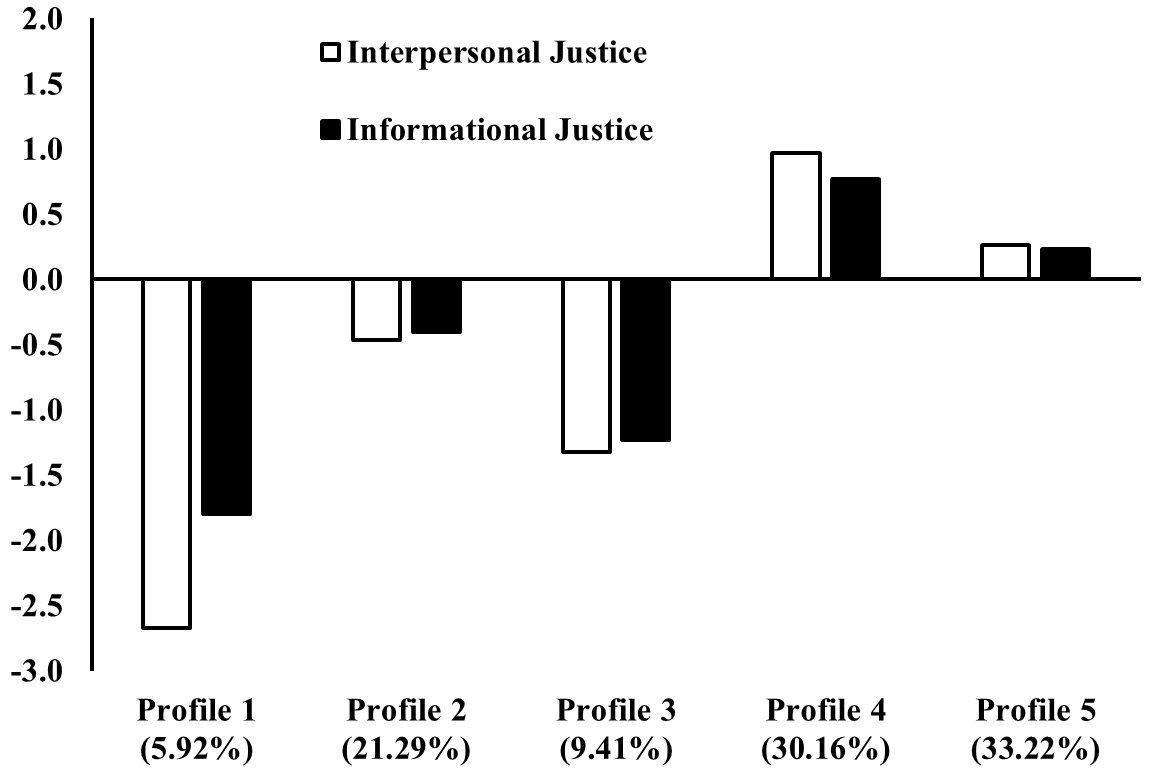
**

**Figure S3.** Final 5-Profile Solution (Two-Factor CFA Factor Scores)

*Note.* Profile 1: Very Low; Profile 2: Moderately Low; Profile 3: Low; Profile 4: High; Profile 5: Moderately High.

**Table S4**

*Detailed Parameter Estimates from the Final LPA Solution*

|  | Profile 1 | Profile 2 | Profile 3 | Profile 4 | Profile 5 |  |
| --- | --- | --- | --- | --- | --- | --- |
|  | Mean [CI] | Mean [CI] | Mean [CI] | Mean [CI] | Mean [CI] | Variance [CI] |
| *Profile Indicators: Two-Factor CFA* |  |  |  |  |  |  |
| Interpersonal Justice | -2.671 [-2.846; -2.496] | -.463 [-.499; -.426] | -1.327 [-1.471; -1.182] | .972 [.964; .980] | .266 [.253; .279] | .032 [.026; .037] |
| Informational Justice | -1.799 [-1.958; -1.639] | -.402 [-.488; -.316] | -1.234 [-1.371; -1.096] | .768 [.694; .842] | .231 [.165; .296] | .383 [.342; .423] |
| *Profile Indicators:*  *Bifactor-CFA* | |  |  |  |  |  |
| Global Interactional Justice | -1.143 [-1.399; -.888] | -1.579 [-1.738; -1.420] | -.640 [-.928; -.353] | .480 [.418; .542] | -.606 [-.859; -.353] | .379 [.338; .421] |
| Specific Interpersonal Justice | .889 [.618; 1.160] | -1.721 [-1.958; -1.484] | .897 [.537; 1.257] | -.010 [-.062; .042] | -.394 [-1.708; .919] | .495 [.407; .582] |
| Specific Informational Justice | .006 [-.456; .467] | -.178 [-.368; .012] | -1.500 [-1.863; -1.136] | .028 [-.016; .072] | 1.831 [1.046; 2.616] | .301 [.241; .360] |

*Note*. CI = 95% confidence interval. The profile indicators are estimated from factor scores with mean of 0 and a standard deviation of 1. For solutions based on two-factor confirmatory factor analytic (CFA): Profile 1: Very Low; Profile 2: Moderately Low; Profile 3: Low; Profile 4: High; Profile 5: Moderately High. For solutions based on bifactor-CFA: Profile 1: High Interpersonal / Average Informational; Profile 2: Low Interpersonal; Profile 3: High Interpersonal / Low Informational; Profile 4: Normative; Profile 5: High Informational.

**Table S5**

*Classification Accuracy: Average Probability of Membership into Each Latent Profile (Column) as a Function of the Most Likely Profile Membership (Row)*

|  | Profile 1 | Profile 2 | Profile 3 | Profile 4 | Profile 5 |
| --- | --- | --- | --- | --- | --- |
| *Profile Indicators from Two-Factor CFA Model* |  |  |  |  |  |
| Profile 1 | .980 | 0 | 0 | 0 | 0 |
| Profile 2 | 0 | .967 | .012 | 0 | .021 |
| Profile 3 | .009 | .026 | .966 | 0 | 0 |
| Profile 4 | 0 | 0 | 0 | .991 | .009 |
| Profile 5 | 0 | .014 | 0 | 0 | .976 |
| *Profile Indicators from Bifactor-CFA Model* |  |  |  |  |  |
| Profile 1 | .804 | .032 | .043 | .103 | .018 |
| Profile 2 | .025 | .924 | .008 | .035 | .008 |
| Profile 3 | .089 | .004 | .854 | .054 | 0 |
| Profile 4 | .039 | .005 | .010 | .939 | .007 |
| Profile 5 | .032 | .043 | 0 | .090 | .835 |

*Note*. For solutions based on two-factor confirmatory factor analytic (CFA): Profile 1: Very Low; Profile 2: Moderately Low; Profile 3: Low; Profile 4: High; Profile 5: Moderately High. For solutions based on bifactor-CFA: Profile 1: High Interpersonal / Average Informational; Profile 2: Low Interpersonal; Profile 3: High Interpersonal / Low Informational; Profile 4: Normative; Profile 5: High Informational.

**Table S6**

*Results from Multinomial Logistic Regressions for the Effects of Transformational Leadership on Profile Membership (Two-Factor CFA)*

|  | Profile 1 vs. Profile 5 | | Profile 2 vs. Profile 5 | | Profile 3 vs. Profile 5 | | Profile 4 vs. Profile 5 | | Profile 1 vs. Profile 4 | |
| --- | --- | --- | --- | --- | --- | --- | --- | --- | --- | --- |
|  | Coef. (SE) | OR | Coef. (SE) | OR | Coef. (SE) | OR | Coef. (SE) | OR | Coef. (SE) | OR |
| Leadership | -4.152 (.502)* | .016 | -1.291 (.128)* | .275 | -2.534 (.223)* | .079 | 1.196 (.181)* | 3.307 | -5.349 (.535)* | .005 |
|  | Profile 2 vs. Profile 4 | | Profile 3 vs. Profile 4 | | Profile 1 vs. Profile 3 | | Profile 2 vs. Profile 3 | | Profile 1 vs. Profile 2 | |
|  | Coef. (SE) | OR | Coef. (SE) | OR | Coef. (SE) | OR | Coef. (SE) | OR | Coef. (SE) | OR |
| Leadership | -2.488 (.214)* | .083 | -3.731 (.290)* | .024 | -1.618 (.458)* | .198 | 1.243 (.186)* | 3.466 | -2.861 (.476)* | .057 |

*Note*: * *p* < .001; SE: Standard Error of the coefficient; OR: Odds Ratio; The coefficients and OR reflects the effects of the predictor on the likelihood of membership into the first listed profile relative to the second listed profile; Transformational leadership is estimated from factor scores with a mean of 0 and a standard deviation of 1; Profile 1: Very Low; Profile 2: Moderately Low; Profile 3: Low; Profile 4: High; Profile 5: Moderately High.

**Table S7**

*Associations between Profile Membership and the Outcomes (Two-Factor CFA)*

|  | Profile 1  M (CI) | Profile 2  M (CI) | Profile 3  M (CI) | Profile 4  M (CI) | Profile 5  M (CI) | Summary of Significant Differences |
| --- | --- | --- | --- | --- | --- | --- |
| Anxiety | .911 (.633; 1.189) | .038 (-.084; .160) | .524 (.310; .738) | -.167 (-.257; -.077) | -.183 (-.267; -.099) | 1 > 3 > 2 > 4 = 5 |
| Emotional exhaustion | .407 (.140; .674) | .085 (-.042; .181) | .171 (-.027; .369) | -.174 (-.270; -.078) | -.018 (-.116; .080) | 2 = 3= 5 > 4; 1 > 2 = 5> 4; 1 = 3. |

*Note*: M: Mean; CI: 95% Confidence Interval; Indicators of anxiety and emotional exhaustion are estimated from factor scores with a mean of 0 and a standard deviation of 1; Profile 1: Very Low; Profile 2: Moderately Low; Profile 3: Low; Profile 4: High; Profile 5: Moderately High.
